# Angiodiversity and organotypic functions of sinusoidal endothelial cells

**DOI:** 10.1007/s10456-021-09780-y

**Published:** 2021-03-21

**Authors:** Philipp-Sebastian Koch, Ki Hong Lee, Sergij Goerdt, Hellmut G. Augustin

**Affiliations:** 1 Department of Dermatology, Venereology and Allergology, University Medical Center and Medical Faculty Mannheim, Heidelberg University, and Center of Excellence in Dermatology, Mannheim, Germany; 2 Division of Vascular Oncology and Metastasis, German Cancer Research Center Heidelberg (DKFZ-ZMBH Alliance), Im Neuenheimer Feld 280, 69120 Heidelberg, Germany; 3 European Center for Angioscience (ECAS), Medical Faculty Mannheim, Heidelberg University, Mannheim, Germany; 4 Faculty of Biosciences, Heidelberg University, 69120 Heidelberg, Germany

**Keywords:** Angiodiversity, Endothelial cell heterogeneity, Liver vasculature, Sinusoids, Angiocrine signaling

## Abstract

‘Angiodiversity’ refers to the structural and functional heterogeneity of endothelial cells (EC) along the segments of the vascular tree and especially within the microvascular beds of different organs. Organotypically differentiated EC ranging from continuous, barrier-forming endothelium to discontinuous, fenestrated endothelium perform organ-specific functions such as the maintenance of the tightly sealed blood–brain barrier or the clearance of macromolecular waste products from the peripheral blood by liver EC-expressed scavenger receptors. The microvascular bed of the liver, composed of discontinuous, fenestrated liver sinusoidal endothelial cells (LSEC), is a prime example of organ-specific angiodiversity. Anatomy and development of LSEC have been extensively studied by electron microscopy as well as linage-tracing experiments. Recent advances in cell isolation and bulk transcriptomics or single-cell RNA sequencing techniques allowed the identification of distinct LSEC molecular programs and have led to the identification of LSEC subpopulations. LSEC execute homeostatic functions such as fine tuning the vascular tone, clearing noxious substances from the circulation, and modulating immunoregulatory mechanisms. In recent years, the identification and functional analysis of LSEC-derived angiocrine signals, which control liver homeostasis and disease pathogenesis in an instructive manner, marks a major change of paradigm in the understanding of liver function in health and disease. This review summarizes recent advances in the understanding of liver vascular angiodiversity and the functional consequences resulting thereof.

## Introduction

Vascular functional heterogeneity is an ancient concept dating back to Hippocrates (460-370 B.C.) and Galen (129-205 AD) who already believed that the cardiovascular system consists of two independent networks made of arteries and veins [1]. Beyond the first attempts to understand the blood vascular system as a whole, technical advances in microscopy and cell culture have facilitated the characterization of organ to organ (inter-organ) vascular specification [2, 3]. Recent high-resolution studies on organotypic vasculatures have shed insight into the molecular repertoires of different vascular beds. Microarray analyses enabled to decipher organotypically differentiated endothelial cells (EC) identifying differentially expressed transcription factors (TFs) and angiocrine (i.e., EC-derived) signatures regulating organotypic vascular heterogeneity [4]. Yet, the analysis of the intra-organotypic vascular heterogeneity remained elusive, mainly due to technical limitations. Recent advances in single-cell technology have eventually allowed to generate high-resolution epigenomic, transcriptomic, and proteomic maps of blood vessels within different organs. Concurrently, these data help to elucidate novel and distinct molecular markers for both inter- and intra-organ specialization and heterogeneity of differentiated EC [5]. It is increasingly recognized that inter- and intra-organotypically differentiated EC have unique molecular programs and mechanisms to exert highly specialized functions, adapted to the needs of the respective vascular bed within the different organs. Therefore, the organotypic vasculature is no longer perceived as a simple passive conduit to deliver blood. Instead, organotypic EC are now widely recognized as active gatekeeper of their immediate tissue microenvironment capable of secreting EC-derived paracrine-acting cytokines, so-called ‘angiocrine’ factors [6].

This review summarizes the current knowledge of organotypic vascular differentiation focusing on the liver, which has in recent years emerged as prototypic example of organ-specific angiodiversity. The microvasculature of the liver is composed of liver sinusoidal endothelial cells (LSEC) that line the liver sinusoids. LSEC have unique morphological features and are functionally highly specialized. They extend from the portal area and carry blood along the sinus to eventually drain through the central vein into the inferior cava vein, thereby generating physical gradients of oxygen, shear stress, and redox along the axis of the liver lobule. LSEC are specialized according to their location in the sinusoid and categorized into periportal, midlobular, and centrilobular subtype [7]. Recent single-cell RNA sequencing analyses could deconvolute this spatial zonation of LSEC along the axis of the liver lobule in substantial molecular detail [8, 9]. Moreover, demarcated markers for LSEC and hepatocytes from different metabolic zones could be identified in both human and murine liver, allowing to spatially decipher the crosstalk between these cells which indicates functional diversity of LSEC subtypes [8–10]. For example, pericentral LSEC, located adjacent to the central vein, are the main source of the Wnt ligands Wnt2 and Wnt9b as well as the Wnt-signaling enhancer Rspo3. These angiocrine Wnt factors are indispensable in establishing a unique niche, which fuels Wnt-addicted self-renewing Axin2- and Lgr5-positive hepatocytes. Loss of angiocrine Wnt signatures consequently leads to the loss of the central hepatocyte signatures with subsequent perturbation of liver size, liver zonation, and impaired regenerative capacity of the liver [11–16].

Midlobular LSEC represent the major EC subpopulation of the sinusoids. They display lymphatic-like properties expressing scavenger receptors such as CD206, STAB1, STAB2, LYVE-1, and VEGFR3. Indeed, midlobular LSEC serve as scavenger and antigen-presenting cells facilitating the clearance of a variety of factors, immune complexes, viruses, and lipopolysaccharides from the circulation [6]. Moreover, angiocrine Bone morphogenetic protein (BMP)2- and BMP6 signaling by midlobular LSEC controls local and systemic iron metabolism [17, 18].

Periportal LSEC display ‘tip cell’-like features due to the expression of *Dll4* and *Esm1*. They thereby create a unique niche for resident LSEC progenitor cells. A recent publication reported CD157-positive resident endothelial progenitor cells residing adjacent to the portal vein that can undergo proliferative expansion following acute liver damage [19]. Embryonically, portal EC serve as niche for hematopoietic progenitor cells during fetal liver hematopoiesis [20]. Furthermore, Notch signaling in portal EC orchestrates monocyte recruitment and differentiation of Kupffer cells (KC), which are liver-resident macrophages. Depletion of KC results in LSEC and hepatic stellate cell (HSC; liver-specific pericytes) activation, which reprogram to orchestrate monocyte recruitment and further instigate the differentiation of recruited monocytes via Notch-BMP signaling [21]. Moreover, a so-called “immune zonation” along the sinusoids, created by periportal localization of myeloid and lymphoid resident immune cells, was found to result from gut microbiota-induced MYD88-dependent signaling in LSEC to limit inflammatory responses in periportal areas while protecting the pericentral niche [22].

Specialized LSEC are not just involved in maintaining liver homeostasis. They also play pivotal roles in liver regeneration and adaptation to pathological challenge. The loss of organ-specific LSEC differentiation, called sinusoidal capillarization, not only changes LSEC morphology with the loss of fenestrations and synthesis of pro-fibrotic molecules, such as basement membrane collagen IV or laminins [23]. More importantly, it impairs a fundamental LSEC function, namely the capacity to maintain the quiescence of HSC. In the healthy liver, HSCs, which represent the liver cells with the most fibrogenic potential, are quiescent, non-proliferative and store vitamin A (retinol). Upon activation, they switch to a proliferative and inflammatory phenotype producing excess extracellular matrix (ECM) molecules [24]. Sinusoidal capillarization, thereby, represents an essential step in the development of fibrotic liver disease. Acute injury is compensated by the capability of the liver to regenerate, but when liver injury becomes chronic (e.g., in non-alcoholic steatohepatitis (NASH), viral hepatitis, alcoholic liver disease, and autoimmune disorders of the liver), liver fibrosis with excessive deposition of ECM is unavoidable. In all of these settings, LSEC are the first liver cell population to be exposed to toxic stimuli. It is, therefore, not surprising that LSEC dysfunction precedes liver fibrosis [25]. Hence, restoration of LSEC differentiation critically contributes to maintaining HSC quiescence and promotes resolution of liver fibrosis [26].

We will during the first part of this review discuss the unique morphological characteristics of LSEC as well as the molecular drivers and origin of LSEC angiodiversity. The second part will be devoted to the highly specialized functions of LSEC controlling liver homeostasis, regeneration, and disease.

## Angiodiversity of liver sinusoidal endothelial cells (LSEC)

### Anatomical traits of sinusoidal endothelial cells

**Fig. 1:**
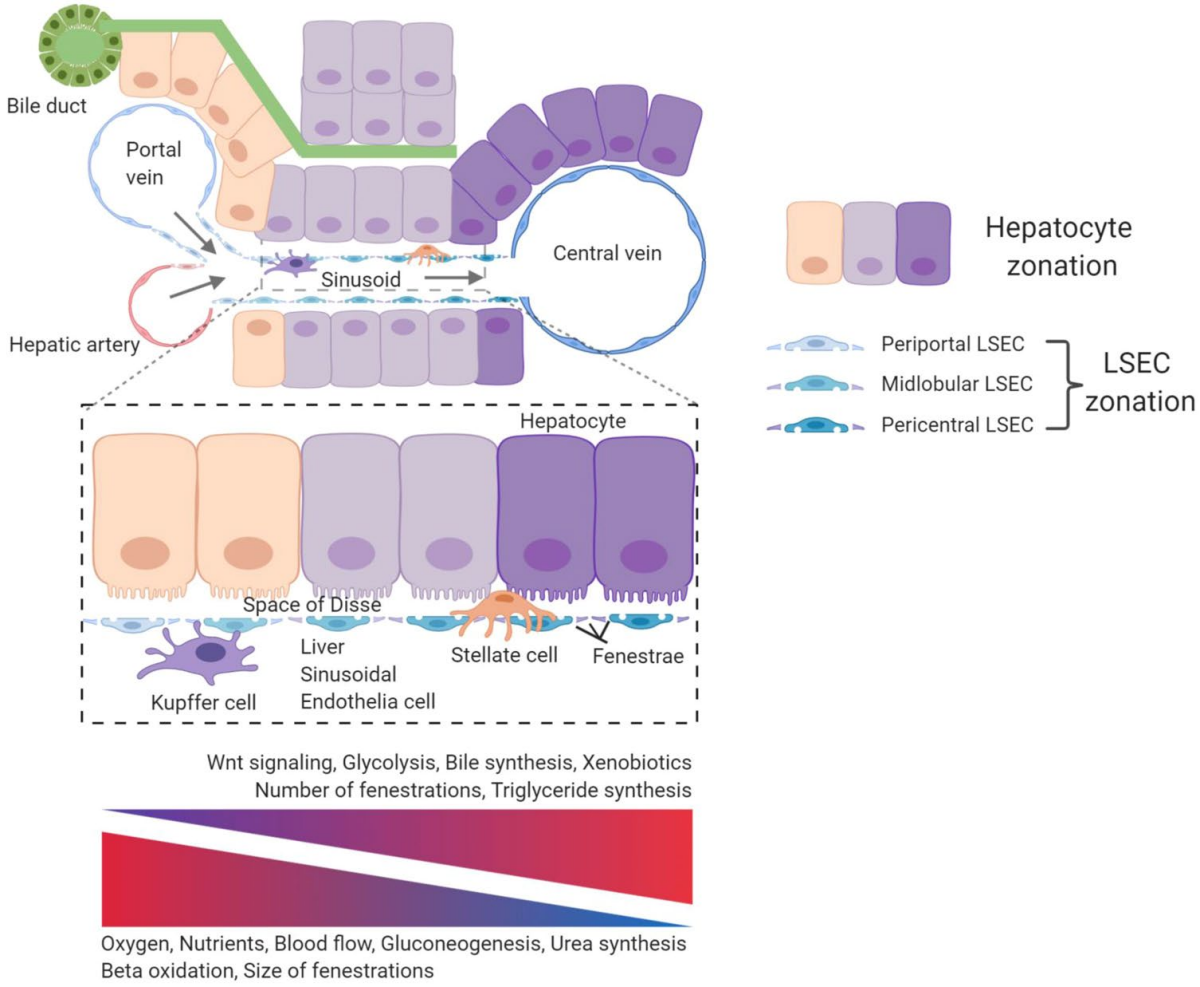
Angiodiversity of the hepatic endothelium. Nutrient-rich blood from the gastrointestinal tract enters the sinusoid via the portal vein and mixes with oxygen-rich blood from the hepatic artery. The blood then moves through the fenestrated sinusoidal vasculature and exits the liver via the central vein creating an oxygen and blood flow gradient along the axis of the liver lobule. These physical gradients establish a “zonation” of hepatocytes and LSEC. Hepatocytes adjacent to the portal vein show high gluconeogenesis, urea synthesis, and beta oxidation activity. In turn, pericentral hepatocytes are characterized with increased glycolysis, bile synthesis, xenobiotics metabolism, and triglyceride synthesis. Fenestrations of LSEC are larger in the periportal area, whereas they are more abundant in pericentral regions. Pericentral LSEC modulate the spatial division of hepatocytes by secreting angiocrine Wnt ligands and Wnt-signaling enhancer Rspo3. The hepatic microenvironment is mainly determined by the interplay of the four major cell populations, namely hepatocytes, LSEC, HSC, and KC. While KC are located in the sinusoids, HSC reside in the space of Disse, which is a perisinusoidal space between LSEC and hepatocytes

The liver vasculature is anatomically and physiologically unique compared to other organs, as the liver has a dual blood supply receiving both, oxygen-rich blood from the hepatic artery (30%) and blood rich in nutrients from the portal vein (70%). After merging, the blood flow continues along the liver sinusoids which are lined by LSEC (Fig. 1). LSEC account for approximately 15–20% of total liver cells and 3% of the total liver mass [6, 27]. They form a discontinuous fenestrated EC layer that is morphologically different from most other EC types due to their unique structure lacking a continuous basement membrane and a diaphragm but instead contain open pores within the EC layer that are called fenestrae [28]. The sinusoidal vasculature enables free transfer of fluid, ions, nutrients, small and large solutes, as well as metabolites to the space of Disse and is thereby in direct contact with the parenchymal liver cells (hepatocytes) and non-parenchymal cells such as HSC (Fig. 1). Beyond the liver, sinusoidal EC are also found in the spleen, the bone marrow, the adrenal medulla, and the pituitary gland [6]. Among these, LSEC remain the best studied type of discontinuous fenestrated EC.

LSEC fenestrae with a diameter of 50-150 nm are grouped in clusters that form a sieve plate. They are highly plastic and dynamic. Intracellular microfilaments, intermediate filaments, and microtubules enable the dynamic adaptation to changing microenvironmental milieus [29, 30]. Periportal and pericentral LSEC exhibit varying degrees of fenestration. Periportal LSEC have lower porosity of fenestrae (larger in size, but fewer in number) compared to pericentral LSEC that contain smaller size fenestrations but are more abundant in number [7, 30]. Likewise, high-resolution analysis revealed the dynamic nature of opening and closing sinusoidal fenestrations in response to varying stimuli [31].

### Development of liver sinusoidal endothelial cells and their self-renewal

**Fig. 2:**
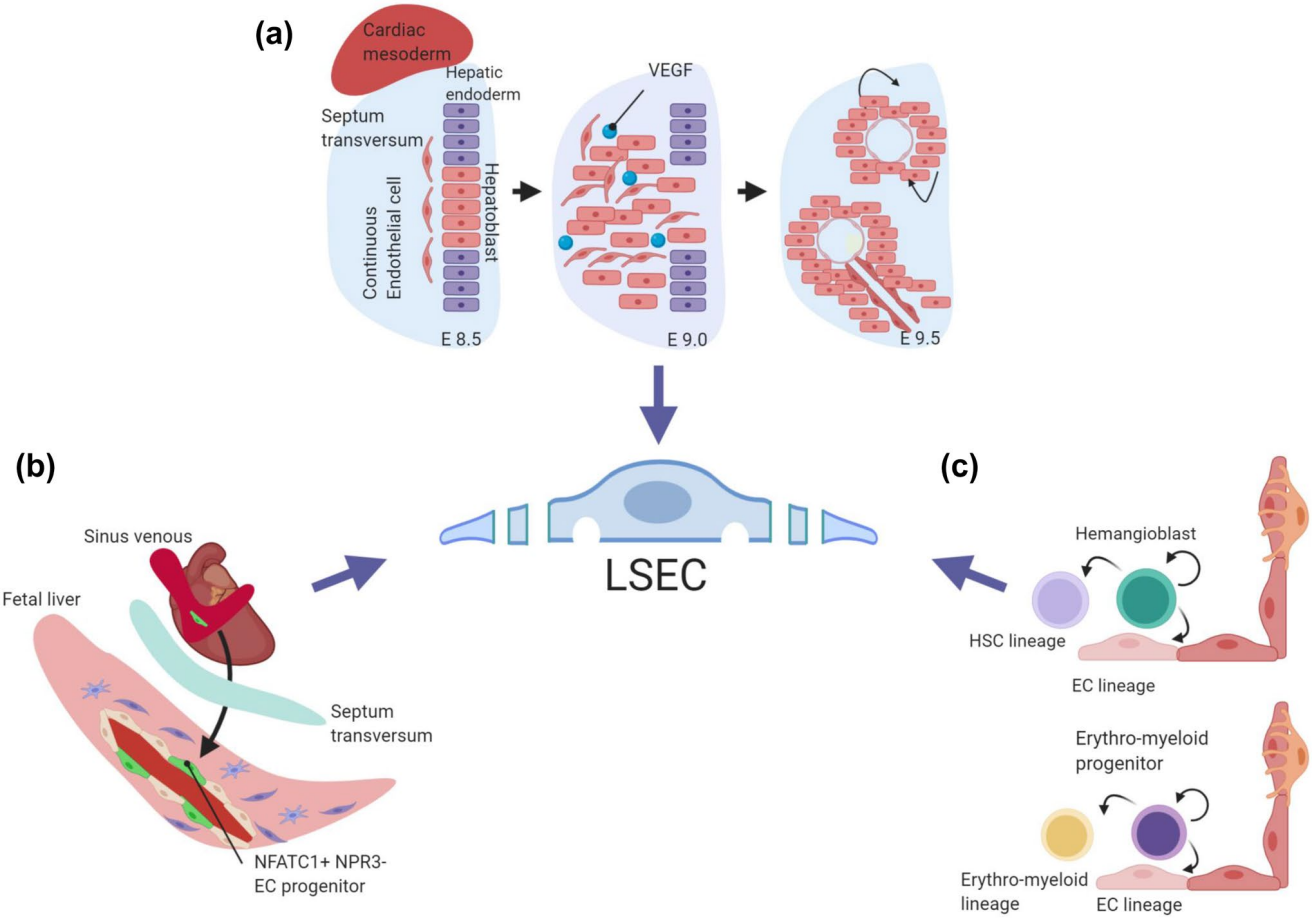
Development of liver sinusoidal endothelial cells. Multiple source may give rise to liver EC, but a unifying concept of LSEC specification is still missing. **a** Genetic in vivo studies have shown that hepatoblasts in the septum transversum mesenchyme coordinate LSEC development through the VEGF signaling pathway. **b** Recent fate mapping studies have indicated a pivotal role of cardiac endothelial progenitor cells in establishing the liver vasculature. Sinus venous-derived NFATC1+ and NPR3- endothelial progenitors may contribute to liver endothelium. **c** LSEC may also be derived from hemangioblasts and/or erythro-myeloid progenitors

Developmentally, LSEC develop from continuous EC. In the human embryo, the phenotypic switch from the continuous to the sinusoidal EC phenotype occurs between 5 and 12 weeks of gestation and is characterized by the loss of continuous EC markers and a reduction of perisinusoidal laminin deposition. Functional LSEC specification is completed around 20 weeks of gestation [32]. Murine LSEC precursors (angioblasts) can first be detected around embryonic day (E)9.0, and the first nascent sinusoids are detected between E9.5 and E10.5 [33] (Fig. 2a).Yet, the transcriptional signatures of sinusoidal EC can be captured as early as E8.75 [34]. Following colonization of the liver by yolk sac-derived stem cells, expansion of hematopoietic stem and progenitor cells (HSPC) starts in the fetal liver around this time of development [35]. The proper switch from continuous to sinusoidal EC specification is critical already during these early stages of embryonic development. Notably, genetic inactivation of the transcription factor *Gata4* in liver EC causes defects in sinusoidal EC specification around E10.5–E11.5. This results in liver hypoplasia, fibrosis, and impaired colonization of HSPC into the fetal liver, leading to anemia and embryonic lethality [36]. These findings have unambiguously established the critical role of hepatic microvascular specification for fetal hematopoiesis and liver development.

These genetic experiments have shown that vascular and organ development are tightly coupled. Indeed, following hepatic specification and liver diverticulum formation, hepatoblast-derived VEGF drives angioblast specification, whereas in turn, differentiating EC are critical for hepatic development prior to forming functioning blood vessels (Fig. 2a). Correspondingly, loss of EC in *Flk1/Kdr* (*Vegfr2*) mutant embryos resulted in defective liver morphogenesis, as cells from the hepatic endoderm could not populate the septum transversum mesenchyme, indicating a critical role of cell-cell interactions between the EC and the hepatic endoderm during liver development [33]. Vice versa, hepatoblasts induce LSEC specification in a paracrine manner via both, direct interaction and VEGF signaling [32, 33, 37]. Similarly, autocrine/paracrine-acting Notch and transforming growth factor β (TGF-β) pathway signaling play pivotal roles during LSEC development [34].

Recent lineage-tracing studies on LSEC development have shown that NFATC1^+^ and NPR3^-^ progenitor cells from the sinus venous endocardium serve as a relevant source of the liver vasculature including LSEC [38] (Fig. 2b). Interestingly, while EC are generally considered to be derived from mesodermal progenitor cells, FOXA2-positive hepatic endoderm cells have also been proposed to give rise to a subset of liver EC [39], suggesting that LSEC can potentially originate from both, mesenchymal and endodermal progenitor populations and even partially share a common progeny with cardiac EC [38, 39]. In zebrafish, the liver vasculature has also been reported to be derived from angioblasts residing in the posterior cardinal vein without arterial contribution [40]. Together, these lineage-tracing experiments indicate different sources for liver EC with LSEC originating from venous derivates [38–40].

There is also evidence suggesting that EC may be derived from hemangioblasts, which represent a common progenitor of the endothelial and the hematopoietic lineages [41, 42] (Fig. 2c). Subsequently, the divergence of sinusoidal and vascular EC from the common EC progenitor occurs around E9.5 [34]. A mouse single-cell molecular map during gastrulation and mesoderm diversification validated the origin of the hematoendothelial lineages with the expression of *Flk1/Kdr* and *Tal1* as molecular switch determining lineage fate to either EC or hematopoietic cells during mesoderm diversification [43, 44]. Hematopoietic cells then further develop and are maintained by the niches within the developing liver, typically by EC and HSC through paracrine-acting stem cell factor [45]. Temporally restricted lineage-tracing experiment of *Cdh5*-expressing cells demonstrated the contribution of common progenitor cells to the liver vasculature [46]. In parallel, myeloid lineage progenitor cells contribute to the liver vasculature during proliferation [47, 48]. Specifically, lineage-tracing experiments with CSF1R, a specific marker for the myeloid lineage, showed incorporation of *Csf1r*-derived LSEC in the liver vasculature demonstrating that LSEC may also be derived from erythro-myeloid linage progenitors (EMP) [48] (Fig. 2c). Yet, this hypothesis was recently challenged as no contribution of EMP cells to the vasculature was demonstrated in independently performed inducible lineage-tracing experiments [49].

Substantial effort has been made to decipher the molecular mechanisms of LSEC differentiation from embryonic (ESC) and pluripotent stem cells (PSC). KDR-positive hepatic progenitor cells may emerge from KDR-negative human ESC in hepatic differentiation medium. These differentiated KDR-positive cells then contribute to the liver EC lineage [50]. Using a murine ESC model, EC derived from ESC have been shown to phenocopy LSEC features when exposed to adrenomedullin and upon inhibition of TGF-β signaling [51, 52]. These findings were also validated in human-induced pluripotent stem cells (iPSC), in which the inhibition of TGF-β in iPSC-derived EC led to a switch to LSEC-like cells [53]. Interestingly both, hPSC-derived arterial and venous angioblasts are capable to differentiate into LSEC-like cells that upregulate LSEC markers and behave functionally as LSEC. Yet, upon balancing cyclic AMP, hypoxia, and inhibition of TGF-β signaling, angioblasts from venous origin upregulate LSEC markers more robustly than angioblasts with arterial pre-differentiation. These hPSC-derived LSEC could be transplanted and exhibited functional LSEC competence [54]. These findings will guide future research aimed at exploring opportunities to *ex vivo* generate and expand functional LSEC for regenerative medicine purposes.

**Fig. 3:**
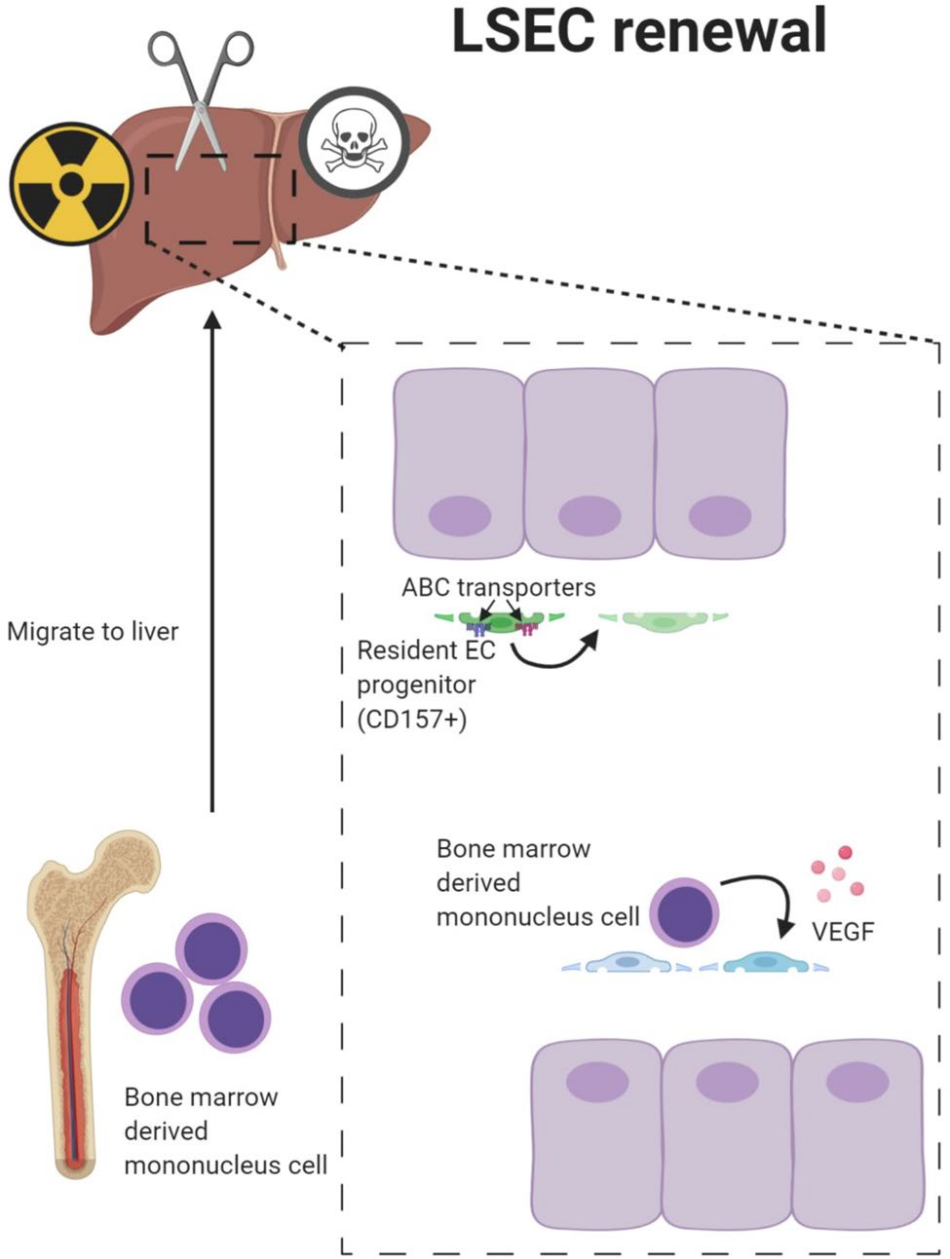
Self-renewal of liver sinusoidal endothelial cells. LSEC are highly plastic and can self-renew upon different challenges. Resident LSEC progenitors have a unique molecular signature expressing CD157 and ABC transporters. CD157-positive LSEC are self-renewable and can replenish the liver microvasculature following challenge. In addition to resident LSEC progenitors, BM-derived progenitor cells may be recruited to the liver and contribute to the regenerating liver vasculature following severe, resident EC damaging challenge such as irradiation-induced vascular injury.

LSEC plasticity during liver damage depends on the fitness of the resident vasculature. Using definite fate mapping techniques, a recent study has shown that liver neoangiogenesis is mediated by the expansion of resident LSEC upon different pathological challenges [55]. Resident LSEC progenitors residing adjacent to the portal vein have been characterized as CD157-positive, self-renewable, ABC transporter expressing cells with stem cell-like features. Upon lineage-tracing, transplantation, and regeneration experiments, CD157-positive cells regenerate the liver vasculature [19] (Fig. 3). Yet, resident LSEC progenitor cells may not be the sole source of the regenerating LSEC vasculature. Bone marrow (BM)-derived progenitor cells have also been proposed to contribute to LSEC regeneration [56, 57] (Fig. 3). Importantly though, bone marrow transplantation in such fate mapping experiments mostly requires radiation, which in itself may lead to massive damage of LSEC. Fate mapping without radiation, e.g., in parabiosis experiments could solidly exclude the contribution of BM-derived progenitor cells towards vascularization during liver regeneration in a partial hepatectomy model [55].

### Transcription factors regulating LSEC differentiation

Microarray analyses of the organotypic sinusoidal vasculatures of the bone marrow and the liver, and to a lesser extent also the spleen, revealed high levels of the Ets TF family member *Sfpi1* [4]. In contrast, sinusoidal *Tbx3* expression was found reduced compared to other vascular beds. Comprehensive gene expression analysis, comparing freshly isolated LSEC with cultured LSEC and rat lung microvascular EC, identified *Gata4* in a cluster of transcriptional regulators (*Gata4, Lmo3, Tfec, Maf*) as one of the critical TF for LSEC differentiation [58]. Indeed, GATA4 is essential for fetal LSEC specification acting as a counter regulator of continuous EC gene expression and inducer of LSEC-specific genes. LSEC-restricted deletion of *Gata4* using *Stab2*-iCre as Cre driver for early embryonic excision resulted in transdifferentiation of sinusoidal EC (STAB2^+^, LYVE-1^+^, CD31^lo^) to acquire traits of continuous capillaries (CD31^+^, EMCN^+^, CAV1^+^) with ectopic basement membrane deposition and increased VE-cadherin expression. This transdifferentiation did not only cause liver hypoplasia and increased ECM deposition, but also impaired immigration of HSPC into the fetal liver resulting in anemia and embryonic lethality. These genetic experiments validated GATA4 as a molecular master regulator of hepatic angiodiversity, controlling LSEC specification and fetal liver development by establishing a hepatic niche required for proper HSPC [36]. Corresponding cellular experiments showed that GATA4 prevents, in cooperation with the transcriptional co-regulator LMO3, the autocrine induction of a pro-inflammatory phenotype while maintaining angiocrine signaling through the GATA4-downstream target BMP2 [59]. Interestingly, ectopic *GATA4* overexpression in HUVEC resulted in the strong suppression of a continuous EC gene signature with a less stringent upregulation of LSEC-associated genes [36].

To bypass early embryonic lethality due to anemia when using *Stab2*-iCre to delete *Gata4* in LSEC, recent experiments have employed *Clec4g*-iCre for late embryonic deletion of *Gata4* in LSEC (around E17.5) [60]. This experimental approach similarly led to induced LSEC-to-continuous EC differentiation including formation of a solid basement membrane resulting in liver fibrosis and hepatopathy in adult mice. *Gata4*-deficient LSEC started to express continuous EC genes as well as angiogenesis-related and pro-fibrotic gene signatures including *Myc*, *Pdgfb,* and *Vegfa* [61]. These experiments also showed that *Gata4* deficiency in LSEC led to a de-repression of general (continuous) EC differentiation, which was mediated by increased chromatin accessibility when *Gata4* was deleted. In turn, no changes on the chromatin level were seen in genes that were downregulated upon *Gata4* deletion indicating that GATA4 has a strong repressive function on continuous EC differentiation on the chromatin level, whereas the activating functions are independent from alterations in chromatin accessibility [61]. Recent murine single-cell data from different vascular beds confirmed that *Gata4* expression and the GATA4 transcriptional network are highly specific for hepatic EC [62].

As GATA4 is not sufficient to fully convert continuous EC into LSEC in vitro [36], other transcriptional regulators or angiocrine factors and microenvironmental regulators may be required to fully guide LSEC specification. Recent TF screenings for LSEC specification identified a combination of C-MAF, GATA4, and MEIS2 to induce LSEC signature genes in vitro such as *CLEC4M, MRC1, LGMN, GPR182, PLXNC1, and SLCOA1* [63]. The study of human iPSC differentiating towards the LSEC lineage identified, based on motif analysis, additional potential TF candidates for LSEC differentiation including IRF2, ERG, MEIS2, SPI1, IRF7, WRNIP1, HIC2, NFIX_NFIB, BATF, and PATZ1, awaiting further in vitro and in vivo functional validation [64]. Similarly, functional LSEC-like cells could be generated from human pluripotent stem cells by a combination of hypoxia, cyclic AMP agonists, and TGF- β inhibition. Venous pre-differentiation proved to be superior to support the expression of LSEC markers and functions than arterial pre-differentiation. Interestingly, venous angioblasts were able to engraft and differentiate in transplantation experiments into LSEC-like cells with expression of CD31, CD32b, and LYVE-1, formation of fenestrations and scavenging activity [54].

## Functional attributes of sinusoidal endothelial cells in health and disease

### Blood flow and dynamics

Within the hepatic sinusoids, LSEC are in direct contact to the circulating blood. They sense changes in blood pressure and shear stress in order to maintain low portal blood pressure to avoid microcirculatory dysfunction. LSEC cooperate with HSC to modulate vascular tone and contraction, producing and releasing vasoconstrictive cyclooxygenase 1 (COX1), thromboxane A2 (TXA2), and endothelin-1 (ET-1), as well as vasodilative nitric oxide (NO) and prostacyclin (see [65] for recent review). Interestingly, the transcription factor Kruppel-like factor (KLF)2 was shown to be induced in hepatic EC by increased shear stress controlling the upregulation of NO, while downregulating vasoconstrictive ET-1 [66]. Thus, it is well established that LSEC respond to mechanical stretch; however, the mechanisms of mechano-sensing and transduction of LSEC are not well studied. During development, the growth of the liver is highly dependent on blood perfusion, which activates β1 integrins and VEGFR3 leading to hepatocyte growth factor (HGF) secretion. In the adult liver, mechano-transduction is sufficient to exert angiocrine HGF-signaling regulating proliferation and survival of hepatocytes. This indicates that balanced blood perfusion is critical for organ growth and also maintenance in the adult through angiocrine signaling mechanisms [67].

During aging, molecular changes in the cells of the liver sinusoids with diminished vasodilatory capacity resulting from reduced expression and phosphorylation of endothelial nitric oxide synthase (eNOS) or reduced NO bioavailability lead to elevated hepatic vascular resistance and increased portal blood pressure [68]. The aged hepatic vasculature strongly upregulates p16, a hallmark of cell senescence [69, 70]. Senescent LSEC show transient upregulation of the scavenger receptors followed by a dramatic reduction of their clearance function during aging. Yet both, long-term and acute inactivation of LSEC p16 expression induces fibrosis [69]. Conversely, short-term ablation of p16 expressing LSEC reduced steatosis and inflammation in a murine NASH model [70].

Microcirculatory dysfunction is a hallmark of chronic liver disease and portal hypertension. Mechanosensitive angiocrine factors released by LSEC have been identified suggesting that portal hypertension may be promoted by neutrophil extracellular trap formation and development of microthrombi in the sinusoids. Mechanistically, stretching of LSEC resulted in the integrin-mediated activation of Piezo cation channels modulating Notch 1 activity. This led to the expression of neutrophil chemotactic chemokine CXCL1 via Hes/Hey nuclear translocation [71]. This newly identified mechanocrine pathway potentially offers novel therapeutic targets for treating portal hypertension, which is linked to liver failure and cirrhosis.

The transcription factor ERG contributes to maintaining an anti-thrombogenic environment in the microvasculature of the liver and the lungs. ERG acts in vascular beds with low shear stress as a chromatin opener for KLF2-dependent regulation of anticoagulant thrombomodulin expression [72]. But LSEC do not just modulate coagulation locally. They are also involved in systemic regulation of platelets and vascular thrombosis via Toll-like receptor (TLR)-2 expression. Recently, the microbiota-triggered expression of TLR2 by liver EC was identified as a novel innate immune signaling pathway regulating hepatic von Willebrand factor synthesis and thereby supporting arterial thrombus formation [73].

### Local and systemic clearance functions

**Fig. 4:**
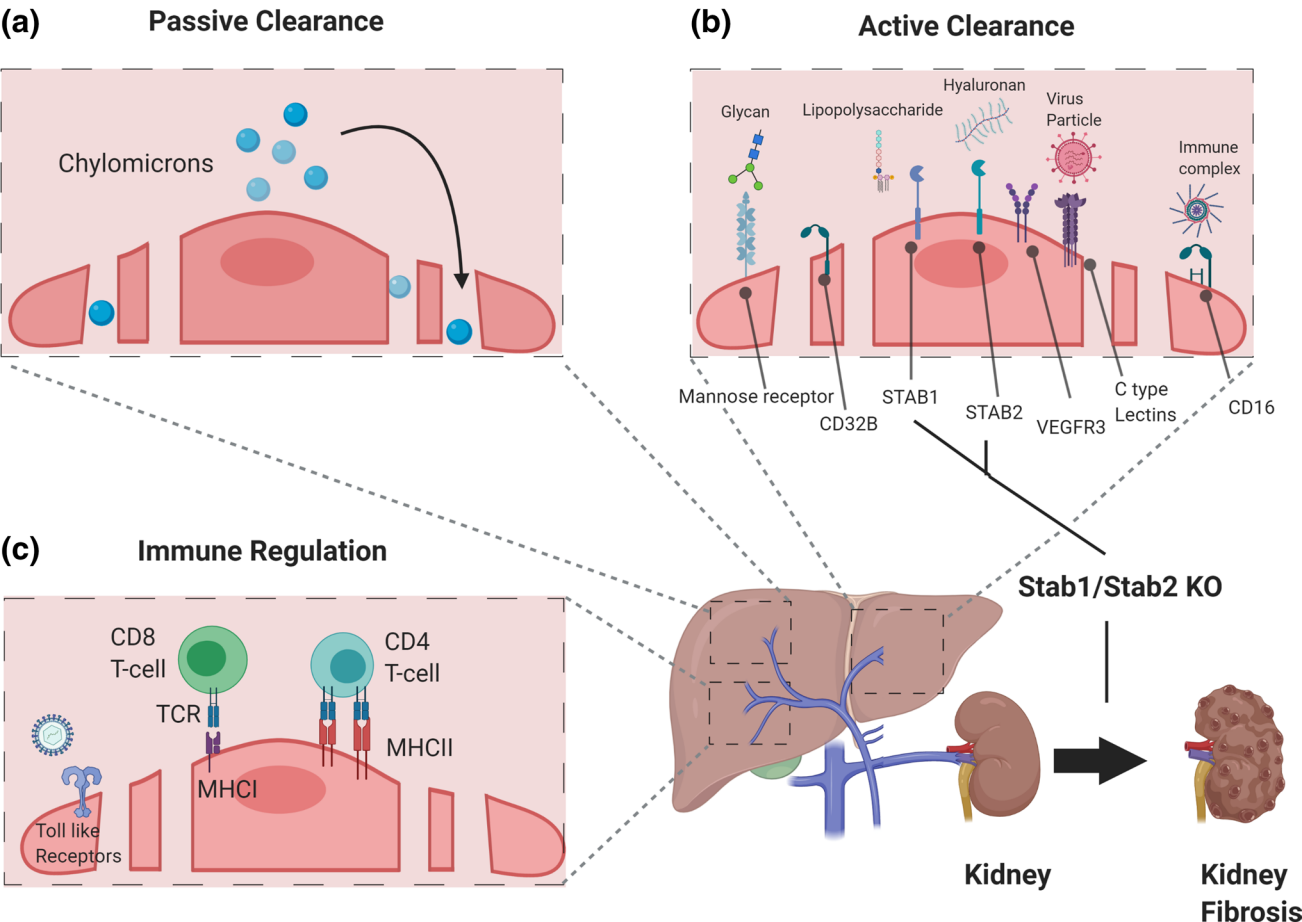
Clearance and immunoregulatory functions of liver sinusoidal endothelial cell. LSEC have distinct organotypic functions capable of acting as professional scavengers and immunomodulators. **a** LSEC-lined sinusoids serve as conduits for the passive transfer of nutrients and soluble factors including circulating chylomicrons through open fenestrations. **b** LSEC execute active scavenger functions clearing pathogens, macromolecules, and waste products through expression of scavenger receptors such as mannose receptor, CD16, CD32b, Stabilin-1, Stabilin-2, VEGFR3, and C-type lectins. **(C)** LSEC play key roles in innate immunity by expressing Toll-like receptors (TLRs). They are also involved in regulating adaptive immunity. LSEC express major histocompatibility complex class I (MHC I) receptors to present antigens to CD8+ T cells and major histocompatibility complex class II (MHC II) receptors to present antigens to CD4+ T cells

Next to passive transport mechanisms, enabled by open fenestrations and the absence of a basement membrane, such as the capacity to filter chylomicron remnants from the blood [74, 75] (Fig. 4a), LSEC are responsible for active clearance of soluble macromolecules, small particles, immune complexes, and lipopolysaccharides (Fig. 4b). In fact, LSEC are among the cells of the body with the highest endocytic capacity. They have numerous micropinocytotic and endocytotic vesicles which may contribute up to 45% of total pinocytic capacity [76]. As the liver serves as the primary detoxification organ of the body, it is essential that LSEC acquire strong lysosomal activity to eliminate waste molecules from the circulation. This clearance is mediated by endocytic and scavenger receptors on LSEC such as the mannose receptor (MRC1/CD206) [77], the Fc gamma-receptor IIb (FcgRII/CD32b) [78], and hyaluronan receptors such as LYVE-1, STAB1, and STAB2 [79] (Fig. 4b). The mannose receptor, which is at much lower abundance also expressed by tissue resident macrophages, binds collagen peptides, tissue plasminogen activator, and lysosomal enzymes for catabolism of endocytosed material [77, 80]. CD32b is responsible for the clearance of IgG-type immune complexes [81]. LSEC-expressed STAB1/2 are involved in the clearance of noxious factors from the circulation, which has not only local, but also systemic effects, such as acting protective for the kidneys [82] (Fig. 4). The stabilins act redundantly as revealed by the observation that mice deficient in either *Stab1* or *Stab2* do no exhibit a major phenotype. In contrast, deletion of both, *Stab1* and *Stab*2, resulted in premature mortality with severe glomerular kidney fibrosis and only mild liver fibrosis and dysfunction. Kidney transplantation from *Stab1/2*-double-deficient mice in wildtype mice prevented glomerular fibrosis, indicating the redundancy of STAB1 and STAB2 in the hepatic clearance of noxious factors from the blood.

### Immunoregulatory functions

**Fig. 5:**
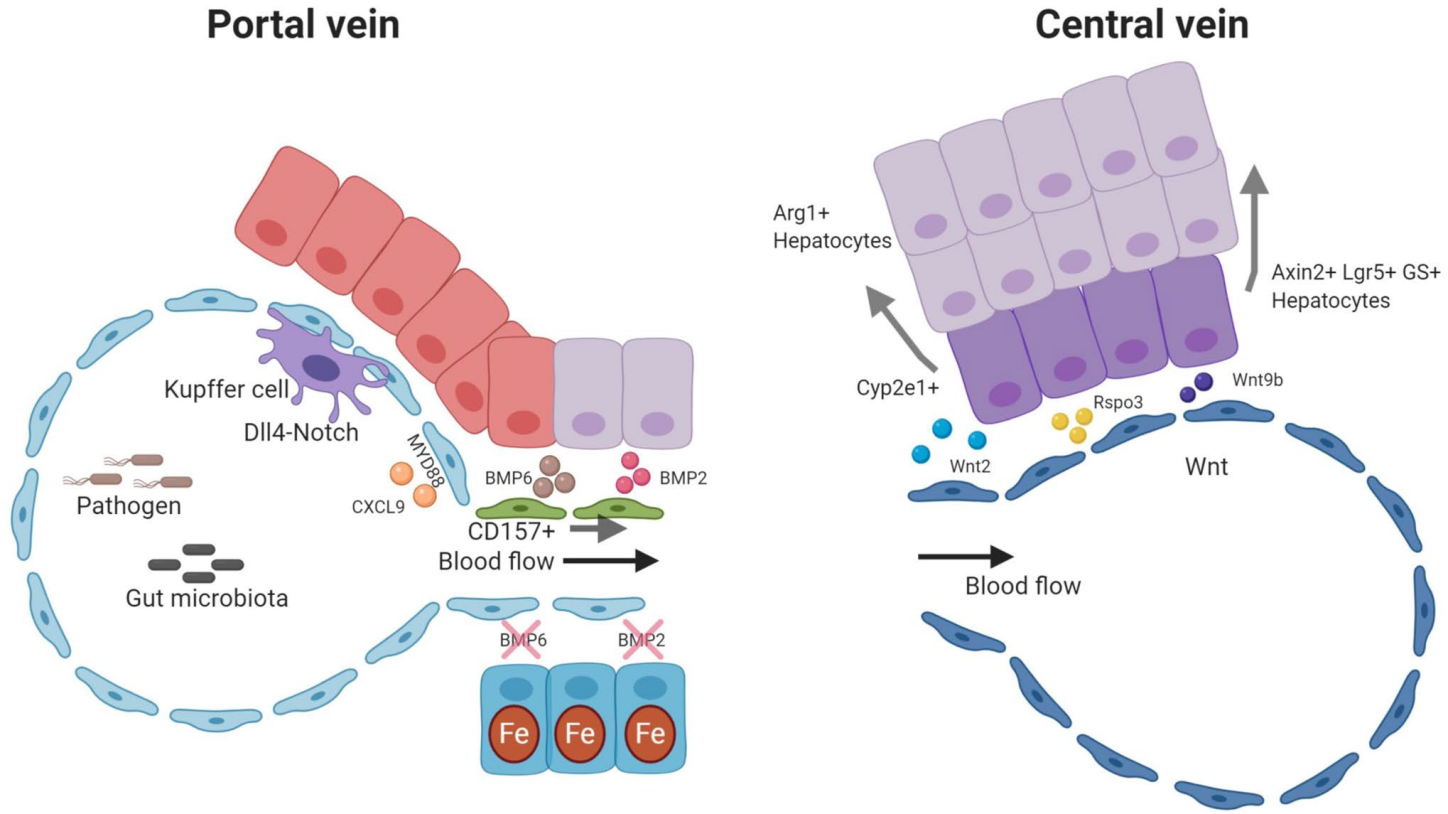
Angiocrine factors control liver function. The hepatic endothelium is not just a passive conduit for blood. Instead, it acts as instructive gatekeeper regulating the hepatic microenvironment through EC-derived angiocrine factors. Pericentral LSEC are a prime example of angiocrine signaling. They modulate hepatocytic function, by secreting angiocrine Wnt2, Wnt9b, and Rspo3 to establish the spatial division of labor of hepatocytes (“metabolic liver zonation”). Concurrently, angiocrine Wnt-signaling fuels Axin2^+^, Lgr5^+^, and glutamine synthase (GS)^+^ pericentral hepatocytes to maintain the pericentral niche during tissue homeostasis. Moreover, angiocrine Wnt-signaling plays a pivotal role in regulating xenobiotic functions by modulating the hepatic cytochrome activity such as CYP2E1. In the portal zone, hepatocytes are actively involved in gluconeogenesis and urea cycle with increased expression of arginase (Arg1). Portal vein EC and periportal LSEC have high Notch activity with upregulated *Dll4* expression. Angiocrine DLL4 may orchestrate monocyte recruitment to establish a niche for KC. Likewise, the hepatic immune zonation is further maintained by LSEC driven CXCL9 and through MYD88 pathway. Periportal LSEC also serve as a niche for resident LSEC progenitors expressing CD157. Midlobular LSEC control iron homeostasis by secretion of the angiocrine factors BMP2 and BMP6

Beyond scavenging of macromolecules, the repertoire of LSEC surface molecules enables regulatory functions in innate and adaptive immunity (see [83] for recent review) (Fig. 4c). Sinusoidal EC are exposed to a large number of gut-derived pathogens, which need to be removed from the circulation. Historically, KC, as liver-resident macrophage population, were considered to serve as the primary cell type responsible for blood clearance. More recently, LSEC gained increasing attention as primary gatekeeper of liver immune functions. Structurally, pathogens from the gut enter into the liver via portal vein forming portal ECs as the front line of host defense. LSEC have the ability to actively sense gut microbiota and control an immune zonation in the liver with periportal positioning of innate and adaptive immune cells to promote host defense, i.e., KC-mediated protection from bacterial dissemination and sepsis in MYD88- and CXCL9-dependent manner. LSEC-specific *Myd88* and *Cxcl9* null mice showed spatial disruption of KC indicating a pivotal role of portal ECs to establish the zone of hepatic immune defense [22]. Likewise, portal EC orchestrate monocyte recruitment and differentiation of KC through Notch-BMP pathway [21] (Fig. 5). Moreover, antigens are cleared by pattern recognition receptors expressed by LSEC. Among these, there are not only the aforementioned scavenger receptors STAB1/2 and MRC1, but also TLR1-4, -6, -8, -9, and C-type lectin (CLEC) receptors (DC-SIGN/CLEC4L, L-SIGN/CLEC4M, LSECTIN/CLEC4G) [83].

LSEC are also highly efficient in clearing viral particles form the blood. DC-SIGN and L-SIGN are involved in the clearance of Ebola virus, Human Immunodeficiency Virus, and Corona viruses [84, 85]. Moreover, Adenovirus 1 and BK/JC Polyoma-like particles have been shown to be rapidly and efficiently cleared by LSEC [86, 87]. Beyond their function as a so-called “*defendothelium*” [88], immunotolerant properties of LSEC are imperative to limit hepatic inflammation and subsequent tissue damage, which may, for example, be lost during viral hepatitis by a shift from an anti-inflammatory towards a pro-inflammatory profile [89]. LSEC-dependent immunotolerance is induced by EC-mediated induction of IL10-producing Th1 cells [90] or Treg [91], or by suppression of Th1 and Th17 cells by IL10 secreted from LSEC which express MHC class II molecules. Vice versa, LSEC that also express MHC class I, are capable of cross-priming naïve CD8+ T cells [92] (Fig. 4c). Mediated by MRC1 receptor, LSEC can take up and process antigens to present them via MHC-I to CD8+ T cells, thereby promoting tolerance controlled by upregulated expression of co-inhibitory programmed cell death ligand 1 (PD-L1) [93, 94]. However, the promotion of tolerance is highly dependent on antigen concentration. While low antigen concentrations lead to tolerance, high amounts of harmful antigens result in cross-priming of CD8+ T cells enabling effector T cell responses [83, 95].

Recruitment of lymphocytes to LSEC is mediated by intercellular adhesion molecule-1, vascular adhesion protein-1 and STAB1 [96] and is further supported by low blood pressure and shear stress within the sinusoids. Beyond transcellular and paracellular migration, lymphocytes adhere to the hepatic endothelium to migrate across and along the LSEC layer, which is further enhanced by interferon-ɣ stimulation [97]. Independent of diapedesis, the uniquely fenestrated morphology of the sinusoids allows T cell foot processes to reach out into the space of Disse. They thereby get in physical contact with MHC-I expressing liver parenchymal cells to immunosurveillance hepatocytes, for example, to eliminate virus-infected cells. The initial arrest of T cells on LSEC may involve the docking to platelet aggregates adhering to sinusoidal hyaluronan bound via CD44. Interestingly, loss of LSEC fenestrae (capillarization) and fibrosis with increased deposition of ECM in the space of Disse impairs T cell immunosurveillance, which might limit viral host defense mechanisms and ultimately promote the development of hepatocellular carcinoma (HCC) [98].

### Vascular control of hepatocyte function

Sieve plate fenestrations of LSEC separate the hepatic blood and the plasma in the space of Disse. These fenestrations enable the bi-directional transport of blood-borne molecules, metabolites, nutrients, and detoxification products to and from hepatocytes and HSC [99] (Fig. 1). The diverse metabolic functions of hepatocytes occur in different functional areas/zones within the liver lobule, whose positioning is controlled by distinct gene expression programs. This heterogeneity of hepatocytes is generally referred to as “*metabolic liver zonation*” (Fig. 1). Perturbation of metabolic liver zonation is regularly seen in non-alcoholic fatty liver disease (NAFLD), NASH, liver cirrhosis, and HCC [100]. While canonical Wnt-β-catenin signaling was identified as the decisive driver of metabolic zonation [101], the cellular origin of Wnt ligands remained unclear for many years. Recently, angiocrine Wnt ligands have received considerable attention. Both, inducible EC-specific deletion of the Wnt secretion mediator Evi/wntless (*Evi/Wls*) as well as the inducible global deletion of the Wnt-signaling enhancer *Rspo3* impair metabolic liver zonation and liver regeneration due to perturbed Wnt signaling in hepatocytes [12–14, 16, 102]. Detailed analysis of the angiocrine signaling mechanisms controlling metabolic zonation showed that Axin2-positive pericentral hepatocyte progenitor cells expand over a period of one year from the pericentral area to the portal vein and replace preexisting trabecular hepatocytes. Inducible partial excision of endothelial *Evi/Wls* caused angiocrine Wnt deficiency as well as loss of normal pericentral *Axin2*-positive, glutamine synthetase (GS)-positive hepatocyte progenitor cells, i.e., loss of liver zonation, accompanied by reduced proliferation of pericentral hepatocytes and reduced liver regeneration. In fact, liver EC-derived angiocrine Wnt signaling controls not only metabolic liver zonation, but also total liver size. In turn, the corresponding intrahepatic EC zonation is not affected by deletion of EC-derived Wnt ligand secretion. Specifically, disruption of *Evi/Wls* from hepatic EC resulted in the loss of pericentral β-catenin target genes such as GS, RhBg, *Axin2,* and cytochrome P450 2E1, and extended the expression of periportal genes such as arginase 1 (Fig. 5). Interestingly, ablation of angiocrine Wnt signaling also altered lipid metabolism by reducing plasma cholesterol levels and increasing total acylcarnitine blood levels which resembles mouse models of β-catenin signaling depletion in hepatocytes [15, 103–106].

Hepatocytes are well known for their ability to store iron. In this respect, another important gatekeeper function of sinusoidal EC was just recently identified with the demonstration that LSEC are a relevant source of BMP2 and BMP6 (Fig. 5). Secretion of LSEC-derived BMP2 and BMP6 controls iron metabolism in hepatocytes in a non-redundant angiocrine manner. Loss of endothelial BMP2 or BMP6 signaling caused severe iron overload in the liver and serum, similar to classic hereditary hemochromatosis, mediated by a reduced hepatocytic hepcidin expression, which is a key regulator of hepatocyte-controlled iron homeostasis [17, 18]. The detailed mechanisms of iron sensing by LSEC, which lead to balanced BMP secretion and consecutive hepcidin expression, remain elusive.

**Fig. 6:**
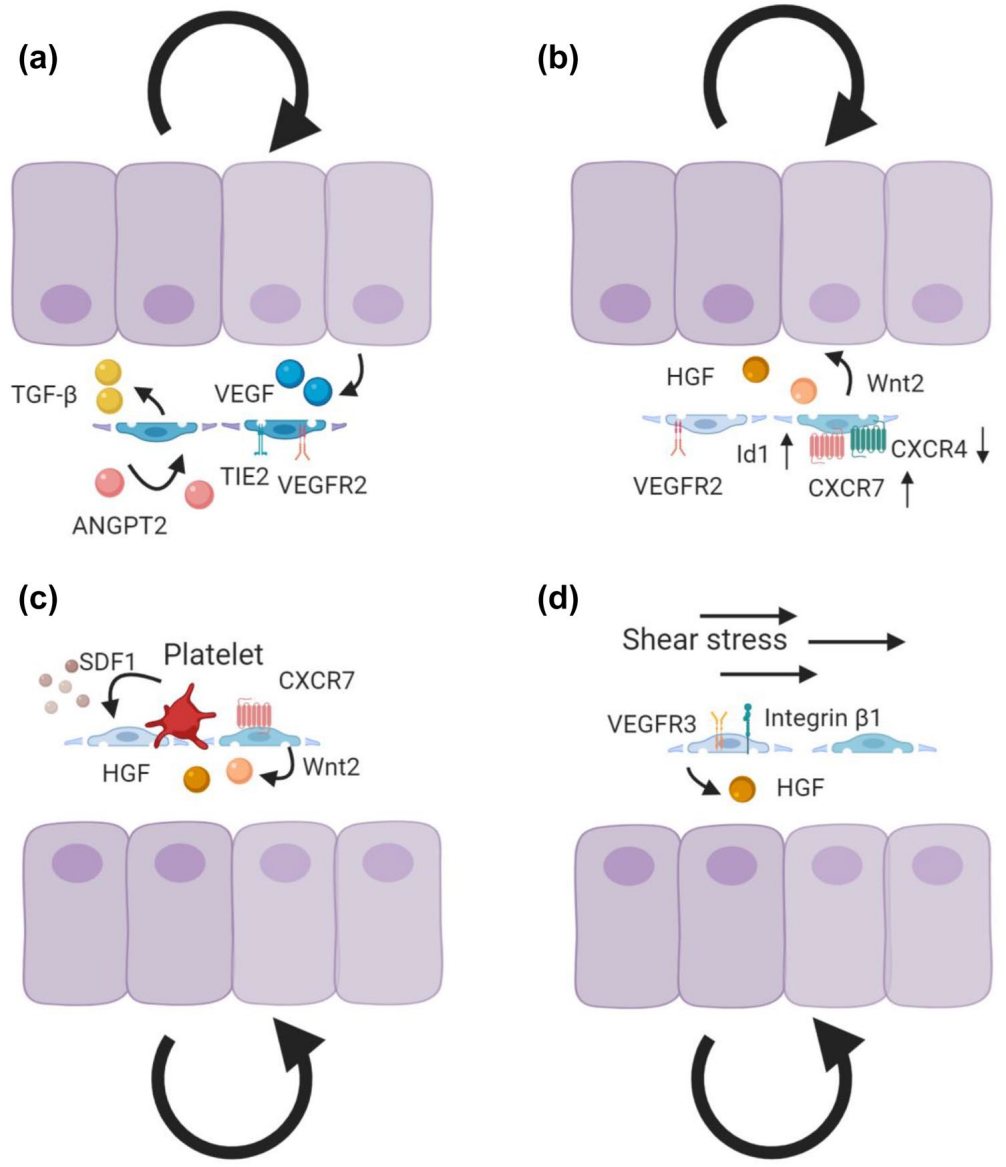
Angiocrine control of liver regeneration. Angiocrine signaling is indispensable for liver regeneration. **a** During the early inductive phase of liver regeneration, downregulation of EC ANGPT2 leads to downregulation of TGF-β production, thereby facilitating hepatocyte proliferation. During the later angiogenic phase, EC ANGPT2 production rebounds to control LSEC proliferation by regulating VEGFR2 in an autocrine Tie2-dependent manner in response to hepatocyte-derived VEGF. EC ANGPT2 thereby serves as a spatiotemporally regulated rheostat dynamically controlling regenerating hepatocytes and angiogenic EC. **b** The balance between pro-regenerative (CXCR7-Id1) and pro-fibrotic (CXCR4) pathways modulates liver regeneration. The pro-regenerative CXCR7-Id1 pathway is upregulated during liver regeneration, to induce Wnt2 and HGF. In addition to CXCR7, the VEGFR2-Id1-mediated pathway triggers the angiocrine factors Wnt2 and HGF to boost hepatocyte proliferation. **c** Activated platelets from the injured area release stromal-derived factor-1 (SDF1) to activate pro-regenerative CXCR7-mediated signaling. **d** Angiocrine HGF can be induced by blood perfusion-regulated mechano-sensing mechanisms. Increased blood flow resulting from hepatic injury upregulates VEGFR3 and β1-integrins for HGF production

The liver is a highly plastic organ with a remarkable capacity of scar-free tissue regeneration after acute injury, which is driven by guided proliferation of hepatocytes and non-parenchymal cells of the liver (Fig. 6). LSEC play an instructive role in regulating liver regeneration via angiocrine VEGFR2-DNA-binding protein inhibitor 1 (Id1)-Wnt2 and -HGF signaling to mediate hepatocyte proliferation [107] and susceptibility to necrosis after partial hepatectomy via HGF/c-Met involving Deptor to prevent excessive organ damage [108].

The Angiopoietin (Angpt)/Tie ligand/receptor system is another vascular signaling pathway that controls liver regeneration through intricate angiocrine signaling mechanisms. During the early inductive phase of liver regeneration (day 0-3) following partial hepatectomy, LSEC expression of ANGPT2 is strongly downregulated resulting in downregulation of LSEC TGF-β production and thereby the removal of an endogenous break of hepatocyte proliferation. The early phase of hepatocyte proliferation is followed by an angiogenic phase of tissue regeneration (4-8 day). During these later stages of liver regeneration, EC restore ANGPT2 expression which facilitates EC expression of VEGFR2 and thereby leads to angiogenic EC proliferation in response to hepatocyte produced VEGF [109] (Fig. 6a). Additionally, stromal-derived factor-1 (SDF1)-receptors CXCR7 and CXCR4 balance regenerative (CXCR7-Id1) and pro-fibrotic (CXCR4) programs. Following acute injury, CXCR7 is upregulated in LSEC to induce regenerative Wnt2, HGF, and angiogenic factors apelin and follistatin-like-1, whereas fibroblast growth factor receptor (FGFR)1-mitogen activated protein kinase-mediated CXCR4 upregulation and perturbation of CXCR7-Id1 signaling occurs after chronic injury [110] (Fig. 6b). Moreover, activated platelets may contribute to liver regeneration by secreting SDF1 to activate the LSEC-mediated regenerative CXCR7 pathway [111] (Fig. 6c). Correspondingly, transcriptional profiling of regenerating LSEC has uncovered regenerating angiocrine signatures such as *HGF*, *Wnt2*, *Angpt2*, *BMP2*, and *MMP*8 [4].

High-resolution mapping enabled to study dynamic transcriptional signatures and interactomes between hepatic cells during liver regeneration. The pseudotime analysis of regenerating LSEC has revealed dynamic states of LSEC activation confirming the imperative role of angiocrine Wnt and HGF/c-Met signaling during liver regeneration [112]. Furthermore, there is evidence that shear stress may induce LSEC-derived angiocrine signals during liver regeneration. As the perfusion pressure increases during the early phase of liver regeneration due to loss of liver mass, the dilation-induced mechano-transduction of LSEC is capable to upregulate integrin β1 /VEGFR3 signaling, which further facilitates HGF production and c-Met signaling [67] (Fig. 6d). Vice versa, shear stress-induced TF expression modulates liver regeneration, as the shear-stress-instructed TF KLF2 in LSEC negatively regulates hepatocyte proliferation through induction of an antiproliferative secretome signature including activin A [113].

### Endothelial dysfunction and sinusoidal capillarization

Endothelial dysfunction contributes to severe liver diseases ranging from alcoholic steatohepatitis and NASH to liver cirrhosis and from hepatocarcinogenesis to liver metastasis. During these disease processes, LSEC transdifferentiate towards a capillary phenotype in a process called “sinusoidal capillarization.” This transdifferentiation process classically involves morphological changes including the loss of fenestrations and the formation of a continuous basement membrane [114]. These morphological changes are associated with a distinct molecular switch in EC marker expression from sinusoidal to continuous EC markers [36]. Sinusoidal capillarization of LSEC also occurs to some extent during aging with a reduction of fenestrations and increased thickening of LSEC resulting in hypoxia. This is referred to as “pseudocapillarization” and is mechanistically linked to age-related insulin resistance and hyperlipidemia [68, 115]***.***

**Fig. 7:**
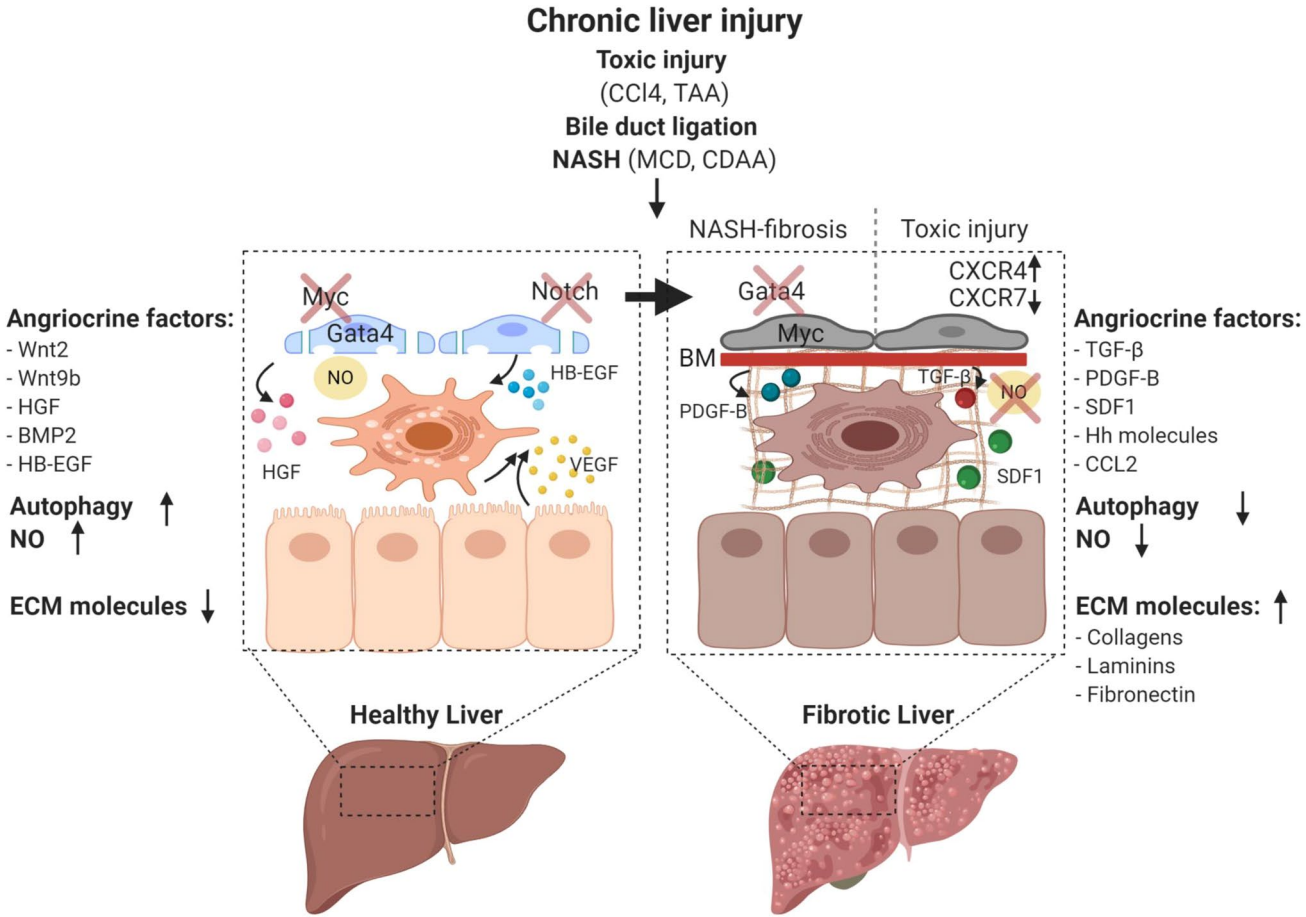
Characteristics of liver sinusoidal endothelial cells during disease progression. Chronic liver damage can be experimentally induced by toxic substances such as carbon tetrachloride (CCl_4_) and thioacetamide (TAA) administration, surgical interventions such as bile duct ligation, as well as dietary models of non-alcoholic steatohepatitis (NASH) including methionine- and choline-deficient diets (MCD) and the choline-deficient L-amino-defined diet (CDAA). LSEC maintain liver homeostasis through nitric oxide (NO) synthesis and secreting angiocrine factors such as Wnt2, Wnt9b, HGF, BMP2, p300 mediated CCL2, and heparin-binding EGF-like growth factor (HB-EGF). The transcription factor GATA4 controls the sinusoidal phenotype including the absence of a basement membrane. During liver homeostasis, autophagic activity of LSEC is increased to protect against liver injury, and there is only little deposition of ECM molecules in the space of Disse. GATA4 is downregulated and continuous EC genes including the transcription factor *Myc* and the angiocrine factor *Pdgfb* are upregulated in NASH-induced perisinusoidal fibrosis. The balance between CXCR7 and CXCR4 shifts and further favors the pro-fibrotic pathways upon toxic liver injury. During fibrosis, angiocrine factors including TGF-β, PDGFB, SDF1, and Hh are dynamically upregulated. Activated LSEC may further trigger HSC to produce excessive ECM. NO bioavailability is lost and the autophagic activity is reduced

The loss of organ-specific endothelial differentiation impairs the capacity of LSEC to maintain HSC quiescence and thereby contributes to HSC activation and fibrotic liver disease (Fig. 7). Hence, restoration of LSEC differentiation results in HSC quiescence and resolution of liver fibrosis [26]. Secretion of VEGF from hepatocytes [37] and HSC is critical for maintaining the fenestrated LSEC phenotype via NO-dependent (VEGF-eNOS-soluble guanylyl cyclase (sGC)-cyclic guanosine monophosphate (cGMP)-protein kinase G) and NO-independent signaling mechanisms [26]. Thus, impairment of the eNOS-sGC-cGMP axis is a major cause of LSEC capillarization [116]. Notch signaling has emerged as another signaling pathway that controls the formation of fenestrae as Notch activation in LSEC results in the loss of fenestrae through altered eNOS-sGC signaling [117]. Moreover, fenestrations can be modulated in a paracrine manner by HSC-derived BMP9 leading to continuous EC differentiation with increased expression of the continuous EC marker CD34 and a reduced number of fenestrae [118].

Hedgehog (Hh) signaling is also involved in phenotypic changes during sinusoidal capillarization. In vitro capillarization of LSEC was associated with the repression of the Hh ligand inhibitor Hhip and induction of Hh downstream targets, evidenced by the finding that smoothened-deficient LSEC showed less capillarization as measured by a reduction of capillarization-associated genes and maintained fenestrations [119]. The liver X receptor (LXRa) transcription factor program was identified as a negative regulator of Hh signaling pathway thereby restoring LSEC differentiation. Capillarization of liver sinusoids was aggravated in a toxic liver fibrosis model in *LXRa*-deficient mice suggesting that LXR agonists are sufficient to maintain LSEC differentiated in vitro [120]. In turn, loss of platelet-derived growth factor (PDGF)B activity increases hepatic permeability, prevents sinusoidal capillarization, and improves insulin sensitivity [121].

### Vascular control of liver fibrosis and cirrhosis

LSEC can integrate external stimuli to act as inductive regulators in fine-tuning physiological (regeneration) and pathological (fibrogenesis) responses to liver injury. Important signaling pathways and angiocrine factors in these processes include the VEGF-SDF1/CXCR4/CXCR7/FGFR1 system [110], the sphingosine1-phosphate receptor-1 (S1P1) [122], ERG via control of the TGF-β-SMAD signaling pathway [123], Notch as a regulator of eNOS-sGC [117], and the HGF/ NADPH oxidase (NOX)4 axis [124]. The signaling pathways during liver fibrosis are complex and interdependent and vary substantially in different experimental models of fibrosis (Fig. 7). Induction of toxic liver injury by administration of carbon tetrachloride (CCl_4_) results in a pericentral type of liver fibrosis with development of centro-portal fibrotic septa, whereas bile duct ligation (BDL) as a surgery-based fibrosis model induces a portal pattern of fibrosis. Dietary mouse models recapitulating features of NASH such as the methionine- and choline-deficient diets (MCD) or the choline-deficient, L-amino acid-deficient diet (CDAA) present with steatohepatitis and a pericellular or perisinusoidal type of liver fibrosis [125].

In toxic liver fibrosis, pericentral hepatocyte damage is amplified by hepatocrine damage signals [126] that directly activate HSC [127] leading to bridging fibrosis between the central vein areas as a response to repair [128]. Capillarized LSEC in toxic liver fibrosis no longer prevent HSC activation [26], but exhibit a pro-fibrotic angiocrine program in LSEC with imbalance in activation from pro-regenerative CXCR7 to pro-fibrotic CXCR4 (FGFR1^+^, CXCR4^+^, TGF-β^+^, BMP2^+^, PDGFC^+^, CXCR7^-^, Id1^-^) that causes proliferation and expansion of Desmin-positive HSC [110]. In line with these findings, Notch activation in LSEC, resulting in sinusoidal capillarization by downregulated eNOS-sGC signaling, led to aggravated fibrogenesis in a CCl_4_-induced toxic liver fibrosis model, most likely due to increased TGF-β-mediated HSC activation [117].

The transcription factor ERG controls TGF-β/BMP-signaling in liver EC to maintain homeostasis and to protect from liver fibrosis. *ERG* deficiency in liver EC results in endothelial-to-mesenchymal transition (EndMT) and spontaneous liver fibrosis by a shift from SMAD1 signaling to pro-fibrotic SMAD2/3 activity. Interestingly, ERG was found significantly downregulated in human fibrotic liver tissues from alcoholic liver disease and primary biliary cirrhosis patients [123]. Activation of endothelial S1P1 by its ligand HDL-bound S1P or S1P-agonist SEW2871 was identified as yet another anti-fibrotic and pro-regenerative pathway, attenuating toxic (CCl_4_) and cholestatic (BDL) liver injury, while promoting functional recovery after partial hepatectomy [122].

Epigenetic mechanisms also impact liver fibrosis. The chromatin-remodeling protein BRG1 in liver EC controls liver fibrosis. EC-specific deletion of *Brg1* decreased ROS production and EndMT leading to attenuation of liver fibrosis upon BDL-induced fibrosis. BRG1, by recruiting histone modifying enzymes, interacts with SMAD3 and AP-1 to induce NOX4 transcription and reactive oxygen species (ROS) production via TGF-β [129]. Interestingly, NOX4 expression in perivascular cells correlated positively with the grade of fibrosis in human liver cirrhosis. As such, NOX4 was induced in perivascular cells of mice with EC-specific deletion of HGF upon partial hepatectomy or CCl_4_-induced fibrosis. A novel angiocrine pathway was thereby identified by which endothelial HGF suppresses perivascular NOX4 in order to stimulate fibrosis-free repair [124]. Furthermore, a pro-fibrotic effect of p300 signaling was demonstrated in LSEC by secretion of monocyte chemoattractant CCL2, which requires the formation of a p300/NFκB/BRD4 activator complex to promote acetylation at the CCL2 enhancer and promoter regions and, thus, may become an interesting target for treatment of portal hypertension and liver fibrosis [130].

Chronic liver injury in rats induced by thioacetamide (TAA) was shown to be associated with the recruitment of putative bone marrow-derived LSEC progenitors (so-called “sproc” cells), which engraft into the liver and contribute to fibrosis but fail to fully differentiate into LSEC due to downregulated VEGF-eNOS-NO-sGC-cGMP pathway signaling under the control of TGF-β, thrombospondin 1 (TSP-1), and a disintegrin and metalloproteinase (ADAM)TS13 [131]. Heparin-binding epidermal growth factor-like growth factor (HB-EGF) was identified in this setting as a relevant angiocrine factor which is shed from the cytosolic membrane of LSEC by ADAMs/MMPs in the normal liver to maintain HSC quiescence [131]. This is further supported by the fact that liver fibrosis is exacerbated in *HB-EGF* KO mice [132, 133].

There is also experimental evidence to suggest that the autophagic activity of LSEC modulates sinusoidal capillarization and LSEC dysfunction by decreasing NO bioavailability and ROS activation. Deletion of the essential autophagy gene *Atg7* in LSEC aggravates liver fibrosis following CCl_4_ challenge by activating HSC potentially via increased oxidative stress while inflammation and liver damage are not remarkably altered [134]. Autophagy has previously been shown to protect LSEC during the early phase of toxic liver injury. Yet, autophagy is also relevant in LSEC during NASH. Notably, a defect in autophagy could be detected in LSEC from NASH patients. Moreover, deficiency of autophagy induced in mice by deleting *Atg5* in EC promoted NASH and liver fibrosis. In contrast to the acute CCl_4_ exposure, high-fat diet as a model of fatty liver disease as well as of administration of CCl_4_ for 6 weeks led to inflammation and liver injury, thereby resulting in increased perisinusoidal liver fibrosis [135].

Fibrosis during NASH starts with perisinusoidal fibrosis and is caused by a cascade of multiple sequential pathogenic hits leading to damage of hepatocytes, but also non-parenchymal liver cells [136]. Among the non-parenchymal cells of the hepatic vascular niche, LSEC play a pivotal role in NASH development [137, 138]. Notably, mice fed a CDAA diet exhibited a reduction in fenestrations within one week of diet prior to the onset of steatosis. These findings are in line with genetic inactivation experiments of the plasmalemma vesicle-associated protein (PLVAP) in mice. Knockout of *PLVAP* led to a reduction of fenestrations resulting in elevated serum levels of triglycerides, LDL, and cholesterol. As a consequence, *PVLAP*-deficient mice spontaneously developed a perisinusoidal type of liver fibrosis as seen in NASH [75].

A recently established single-cell crosstalk map of non-parenchymal cells of Amylin diet-induced NASH revealed a remarkably maintained heterogeneity of LSEC, which could be categorized as periportal, pericentral, and two midzonal LSEC populations. However, LSEC underwent a significant molecular transdifferentiation with increased gene signatures for lipid metabolism, antigen presentation, and chemokine release, whereas genes related to vascular development and homeostasis were downregulated. Moreover, well-known angiocrine factors and receptors such as *BMP2*, *Wnt2*, and *Gpr182* were significantly downregulated in NASH livers [139]. These findings are in line with recent findings showing that LSEC gene signatures including transcription factor *Gata4* are significantly downregulated in a CDAA-diet-induced NASH model with perisinusoidal liver fibrosis, whereas continuous EC genes are induced including MYC target genes and the HSC-activating cytokine *Pdgfb*, which is not expressed by healthy, resting LSEC [61]. Notably, these transcriptomic changes are compatible with alterations in the liver following LSEC-restricted *Gata4* deletion, which results in sinusoidal capillarization, hepatopathy, and perisinusoidal liver fibrosis without changes in hepatic triglyceride levels. *Gata4* deletion in LSEC similarly induced *Myc*, *Pdgfb*, and other pro-fibrotic angiocrine factors such as *Esm1*, *Igfbp5*, and *Sparcl1*, whereas *BMP2* and *Wnt2* were downregulated. Mechanistically, *Gata4* deficiency in LSEC leads to de-repression of continuous EC differentiation by increasing chromatin accessibility allowing MYC-dependent activation of PDGFB. This suggests that downregulation of *Gata4* and its downstream targets is an important pathogenetic factor in CDAA-induced perisinusoidal fibrosis leading to a pro-fibrotic switch in angiocrine signaling [61].

The findings in *Gata4*-deficient mice have been fully confirmed by LSEC single-cell RNAseq data from human cirrhotic livers, which similarly suggest antagonistic functions of GATA4 and MYC with downstream disturbance of the PDGFB/PDGF receptor (R)β signaling axis [140]. This extensive RNAseq analysis identified two disease-specific LSEC clusters in cirrhotic livers, which were defined as *CD34*^*+*^*PLVAP*^*+*^*VWA1*^*+*^ and *CD34*^*+*^*PLVAP*^*+*^*ACKR1*^*+*^. These two clusters co-express pro-fibrogenic genes such as *PDGFD, PDGFB, LOX, LOXL2* and display an immunomodulatory phenotype, enhancing leukocyte transmigration potentially via ACKR1. LSEC from the fibrotic niche actively interact with HSC to promote ECM protein deposition via PDGFR, TNFRSF12A, and Notch signaling pathways. In contrast, “healthy” *CD34*^*-*^*CLEC4M*^+^ EC, which were annotated as LSEC, were reduced in cirrhotic livers [140]. Concurrently, additional EC markers related to EC dysfunction and capitalization were observed during liver fibrosis, such as *Fabp4*, *Pcdh17*, *CD34*, and *Esm1* [141]. While transdifferentiation of LSEC during disease progression is well established, EC heterogeneity is to some extent conserved even during advanced stages of cirrhosis. Notably, pericentral LSEC were shown to be more susceptible to damage with increased signature changes of capillarization compared to EC subpopulations from other regions [142].

Advanced liver fibrosis and cirrhosis are major risk factors for the development of HCC. The angiogenic activity of HCC has been correlated with high risk of microvascular invasion, metastasis, and poor prognosis. Similar to sinusoidal capillarization in liver fibrosis and cirrhosis, transdifferentiation of the sinusoidal vasculature is observed in human and murine HCC with loss of LSEC markers STAB1, STAB2, LYVE-1, CD32b, and GPR182 and induction of the continuous EC marker CD31 [143, 144]. The loss of STAB2 expression in peri-tumorous liver tissue from HCC patients is associated with extended overall survival [143]. These findings correspond well with recent single-cell RNAseq data analyzing EC heterogeneity in livers from HDD-induced and HT-29 tumors. Liver tumor-associated EC did not express LSEC, portal vein EC or central vein EC markers, but a signature related to portal vein EC (*CD63*, *Ehd4*, *CD200*) as well as *Aplnr* and *Col18a1*. Interestingly, EC adjacent to the tumor showed molecular characteristics with an intermediate state between normal LSEC and tumor endothelium [145]. The detailed comparative mouse/human single-cell RNAseq analysis of EC in HCC identified increased *NRP1*, *VEGFR2*, and *PLVAP* expression [146]. Correspondingly, EC from the fetal liver were found to express *PLVAP* and *VEGFR2*, reflecting the oncofetal nature of HCC angiogenesis. Concurrently, FOLR2 expressing tumor-associated macrophages, a gatekeeper of the immunosuppressive milieu, have been detected in both HCC and fetal liver reflecting the tight interplay between EC and macrophages. Indeed, the oncofetal reprogramming is maintained by hepatocyte-derived VEGF and direct cell-to-cell interactions of EC and macrophages via Notch signaling [146].

### Perspectives

The recent advances in cell separation and bulk transcriptomics as well as single-cell RNA sequencing techniques have enabled the identification of distinct LSEC molecular programs and led to the identification of LSEC subpopulations in a hitherto unparalleled level of resolution. As discussed in detail in this review, Id1 [107], LXR-α [120], endothelial Notch signaling [60, 117, 147, 148], KLF2 [113], and ERG [123] act as regulators of differentiated LSEC programs. However, only GATA4 can so far be considered as master regulator of sinusoidal EC specification. GATA4 is non-redundantly required for liver development [36] and to maintain LSEC homeostasis in the adult [61]. The recent identification of the GATA4/MYC/PDGFB/PDGFRβ axis in LSEC as critical regulator of liver fibrogenesis offers a novel targetable pathway for angiotargeted therapies [149, 150]. Unfortunately, upstream mechanisms regulating GATA4 expression in LSEC still remain elusive. Moreover, the molecular programs of LSEC zonation into pericentral, midlobular, and periportal subtypes with a distinct EC marker and angiocrine profile, but also structural and functional differences are still largely unexplored. Future work will have to address which transcription factors are responsible for LSEC zonation. It may well be that different combinations of transcriptional regulators, epigenetic mechanisms, or microenvironmental factors (oxygen and nutrient levels, niche cell heterogeneity) serve in a concerted manner as drivers of intra-organ EC specification and zonation.

While sinusoidal EC in the liver have been extensively studied for their organotypic functions, much less is known about angiodiversity and organotypic functions of sinusoidal EC in other organs. Sinusoidal EC are also found in the spleen, bone marrow, lymphoid tissues, and endocrine organs [6], and transcriptomic analyses revealed molecular similarities, especially for liver, spleen, and bone marrow EC [4, 62]. Similar to the intra-organ angiodiversity in the liver, the BM microvasculature comprises a highly complex system composed of distinct vessel types that were shown to contribute to bone formation and HSPC maintenance and differentiation [151–155]. Not only differences in permeability, but also oxygen levels and flow, between less permeable BM arterial EC, H-type capillary vessels and highly permeable and fenestrated BM sinusoidal EC (SEC) have been proposed to regulate HSPC proliferation and homing [151]. However, the exact role of BM SEC, the most abundant cell type in the BM microenvironment, in HSC maintenance and differentiation remains less well defined as is the influence of distinct vascular niche cells during development and progression of hematological disorders. Of note, exploring the in vivo functions of distinct cell types and cellular subpopulations within the BM microenvironment such as BM SEC remains challenging, especially due to the lack of suitable highly specific Cre-driver mice.

Notch activation in hepatic EC results in sinusoidal capillarization, impaired metabolic liver zonation and aggravation of CCl_4_-induced liver fibrosis, suggesting that Notch inhibition may protect from sinusoidal capillarization [117]**.** While this may provide opportunities for Notch-targeted therapies, contextual organ- and disease-specific functions of Notch signaling must be taken into consideration. In the liver, Notch inhibition promotes hepatic metastases [156] and vice versa, Notch activation protects against liver metastasis of melanoma and colorectal cancer [60]. In contrast, Notch activation in the lung promotes lung metastasis [157].

The better understanding of LSEC angiodiversity may also pave the way towards novel immune-targeted therapies. For example, melittin nanoparticles could recently be shown to modulate LSEC immune functions and thereby suppress liver metastasis [158]. Likewise, the recent breakthrough developments in HCC therapy with the combination of immune checkpoint inhibition (PD-L1) and anti-angiogenesis (VEGF), essentially doubling the therapeutic efficacy of the established standard of care (Sorafenib), ask for a concerted effort aimed at dissecting the mechanism of action of the observed therapeutic synergy, and particularly how and to what extent LSEC angiodiversity contributes to facilitating immune checkpoint-targeted therapy [159]. And beyond cancer, the ability of LSEC to balance defense mechanisms and immunosuppression may lead to novel therapeutic strategies to combat chronic inflammatory and autoimmune-related liver diseases.
